# Vertical and horizontal transmission of plant viruses: two extremes of a continuum?

**DOI:** 10.1038/s44298-024-00030-8

**Published:** 2024-05-20

**Authors:** Lucía García-Ordóñez, Israel Pagán

**Affiliations:** Centro de Biotecnología y Genómica de Plantas UPM-INIA and E.T.S. Ingeniería Agronómica, Alimentaria y de Biosistemas, Universidad Politécnica de Madrid, Madrid, 28223 Spain

**Keywords:** Viral transmission, Viral evolution

## Abstract

Parasites have a variety of mechanisms to be transmitted to new susceptible hosts, which can be largely grouped in two main modes: vertical (i.e., from parents to the offspring) and horizontal (i.e., between hosts regardless of descent). Because between-host dispersal is a key trait for parasite fitness, scientists studying host-parasite interactions have been long interested in understanding the evolution of their transmission mode(s). Most work in this regard has been theoretical, which resulted in the development of the so-called *Continuum hypothesis*. This theory states that because vertically transmitted parasites require the host to reproduce, the evolution of this mode of transmission will involve reduced virulence (i.e., the effect of infection on host fecundity) in order to allow maximal host viable progeny production. Conversely, the evolution of horizontal transmission does not have this limitation and parasites with this mode of transmission will evolve higher virulence. Therefore, a trade-off between both modes of transmission across a continuum of virulence values is predicted, with each transmission mode located at the extremes of the continuum. Using plant viruses as a focal parasite, here we review existing theory surrounding the *Continuum hypothesis* and the experimental work testing the predictions of the theory. Finally, we briefly discuss molecular mechanisms that may explain the existence of vertical-to-horizontal transmission trade-offs and potential implications for the management of virus epidemics.

## Introduction

The capacity to infect new susceptible individuals, that is, the between-host transmission rate, is arguably the most important determinant of parasite fitness, i.e., its capacity to produce a new generation of individuals^1,2^. Owing to its importance, parasites have evolved various mechanisms for between-host dispersal. The vector-borne and trans-mammary infections of plants, humans and other mammals, the egg-borne infections of poultry, the transmission by contact and seed-borne infections of plants, and the transovarial infections of invertebrates are some well (and long) known examples^3,4^. Based on common denominators of these transmission mechanisms, in the 1940s, Gross and others grouped them into two transmission modes: Vertical and horizontal^5^. Under vertical transmission (from here on, VT), parasites are transmitted across generations, and each host can infect only its own progeny. Under horizontal transmission (from here on, HT), parasites are transmitted to all susceptible hosts in the population regardless of descent^6^. Since then, scientists have devoted considerable effort to understanding how one, the other, or both modes of transmission are favored during parasite evolution, to explore the epidemiological consequences of this process, and to analyze the evolutionary forces controlling it. Focusing on plant viruses, here we review: (i) The theory on the evolution of the parasite transmission mode; and (ii) the experimental analyses of the predictions of this theory. Finally, the molecular mechanisms that may explain the relative importance of each mode of transmission through parasite evolution, and the potential implications for the management of epidemics, are also discussed. Although we place particular emphasis on plants viruses, most of what is discussed in this review can be applied to viruses (and to other parasites) at large.

## Transmission modes of plant viruses

To understand how plant viruses evolve HT and/or VT, it is first necessary to summarize how they are transmitted.

Horizontal transmission by arthropods, particularly aphids, is the most frequent and widely studied plant-virus transmission mode, with at least 25 virus genera transmitted this way^7^. Based on the acquisition and inoculation timings, aphid-borne transmission can be divided into non-persistent and persistent. Aphids that transmit plant viruses in a non-persistent manner acquire them within a few seconds. Because of the stylet-dependent nature of non-persistently transmitted viruses such as cucumber mosaic virus (CMV) and turnip mosaic virus (TuMV), aphids remain viruliferous for only short periods and spread mainly over short distances^7^. In contrast, persistently transmitted viruses like begomoviruses or barley yellow dwarf virus (BYDV) require an acquisition period of several minutes/hours but can be retained, and in most cases, remain transmissible, for the vector lifetime^8^. Some persistently transmitted plant viruses can multiply in the vector cells (circulative replicative plant viruses), thus having cross-kingdom host ranges^9^. Soil-borne HT is also a frequent way of plant-virus dispersal. Viruses belonging to at least 17 genera are known to be transmitted by soil-inhabiting organisms^10^, which can be largely categorized into three groups, namely plasmodiophorids (Protista), *Olpidium* spp. (Fungi), and nematodes (Animalia). Some examples of soil-borne viruses are beet necrotic yellow vein virus (BNYVV)^11^, grapevine fanleaf virus (GFLV)^12^ or barley yellow mosaic virus (BYMV)^13^. Plant viruses can also spread by vegetative propagation invading tubers, which is relatively frequent for sweet potato leaf curl virus (SPLCV)^14^ and potato virus Y (PVY) in potato^15^. In addition, plant viruses transmitted by the grafting of infected tissue into a healthy host are not uncommon in grapevine, *Prunus* spp. and citrus tree orchards worldwide^12,16,17^. Finally, mechanical transmission by contact is the major way of dispersal during field epidemics of economically important viruses in the genera *Tobamovirus*, *Potexvirus*, and *Hordeivirus* (e.g., ref. ^18^).

Although less studied, parent-to-offspring VT through seeds has been described for >25% of all known plant viruses^19^ and for some of them, such as persistent (also known as cryptic) viruses, it is the only way to infect new hosts^20^. According to the distribution of the virus in the seed, there are two distinct and non-mutually exclusive mechanisms of seed infection: embryonic (infection of embryos) and non-embryonic (contamination of seeds)^17,19,21–23^. Viruses using an embryonic route are considered seed-transmitted, and most often, the seedlings growing from infected embryos harbor the virus, although not always. For instance, the presence of CMV in pepper seed embryos does not guarantee transmission^24^. Viruses that undergo embryonic seed transmission can infect the embryo by two routes: First, indirectly, by infection of plant gametes prior to fertilization, either the ovules or the pollen^25^. Second, directly from infected maternal tissue, which has been proposed to occur through the embryonic suspensor before its programmed cell death^25^. Plant viruses using a non-embryonic route are thought to be transported externally on the seed coat^26,27^. However, studies revealed that they can also invade the seed coat epidermis and parenchyma cells, and the endothelium that surrounds the endosperm^28,29^. Plant viruses with non-embryonic seed transmission are considered seed-borne because, although they are carried by seeds externally or internally, they do not infect the embryo, and transmission depends on their capacity to infect the seedling during germination^17,30^. The paradigmatic and most studied examples of this type of virus are the members of the genus *Tobamovirus*^27^.

This dichotomy in plant-virus transmission mode opens the question of how these parasites evolve in one and/or the other transmission mode. Moreover, the majority of seed-transmitted viruses can also achieve HT^17^. This is apparently a redundancy that may appear counterintuitive in terms of resource optimization for the virus. Then, why is it so common? These questions have been mostly approached from a theoretical perspective, which is the subject of the next section.

## Theory on the evolution of parasite transmission mode

The importance of parasite (including plant viruses) transmission to understand the emergence and severity of epidemics^31,32^, and the increasing awareness of the role that VT may have in initiating outbreaks and in parasite long-term persistence in the host population^33,34^, has resulted in a well-developed theory on the evolution of parasite transmission modes.

Early mathematical models on the evolution of parasite transmission focused exclusively on HT and its consequences for infection prevalence (see references in ref. ^35^). It was not until the 1970s that Fine^36^ developed what he called the *fundamental vertical transmission equation* with the goal of addressing the contribution of VT to parasite prevalence. Under the assumption that such contribution would be determined by the effect of infection on host progeny production and survival, as well as by the VT rate, the equation predicted that VT parasites would persist in the host population only if infection were beneficial for the host; that is when parasites become mutualists. At any level of virulence (defined as the effect of infection on host fecundity^37^, which in the case of plants is quantified as the number of viable seeds), VT parasites would also require HT for persistence. Applied to virulent plant viruses, these would evolve VT only in co-existence with HT. As a paradigmatic example, this is the case of CMV which can be transmitted either through seeds at low-medium rates or horizontally via aphids^19^. Many other plant viruses follow the same pattern^17^, which would support the prediction of Fine´s model, and then provide a first explanation for why many plant viruses have both transmission modes. This author predicted one exception for this general rule: a (virulent) VT parasite would persist in the host population with no need for HT only at high rates of vertical transmission^36^.

**Fig. 1:**
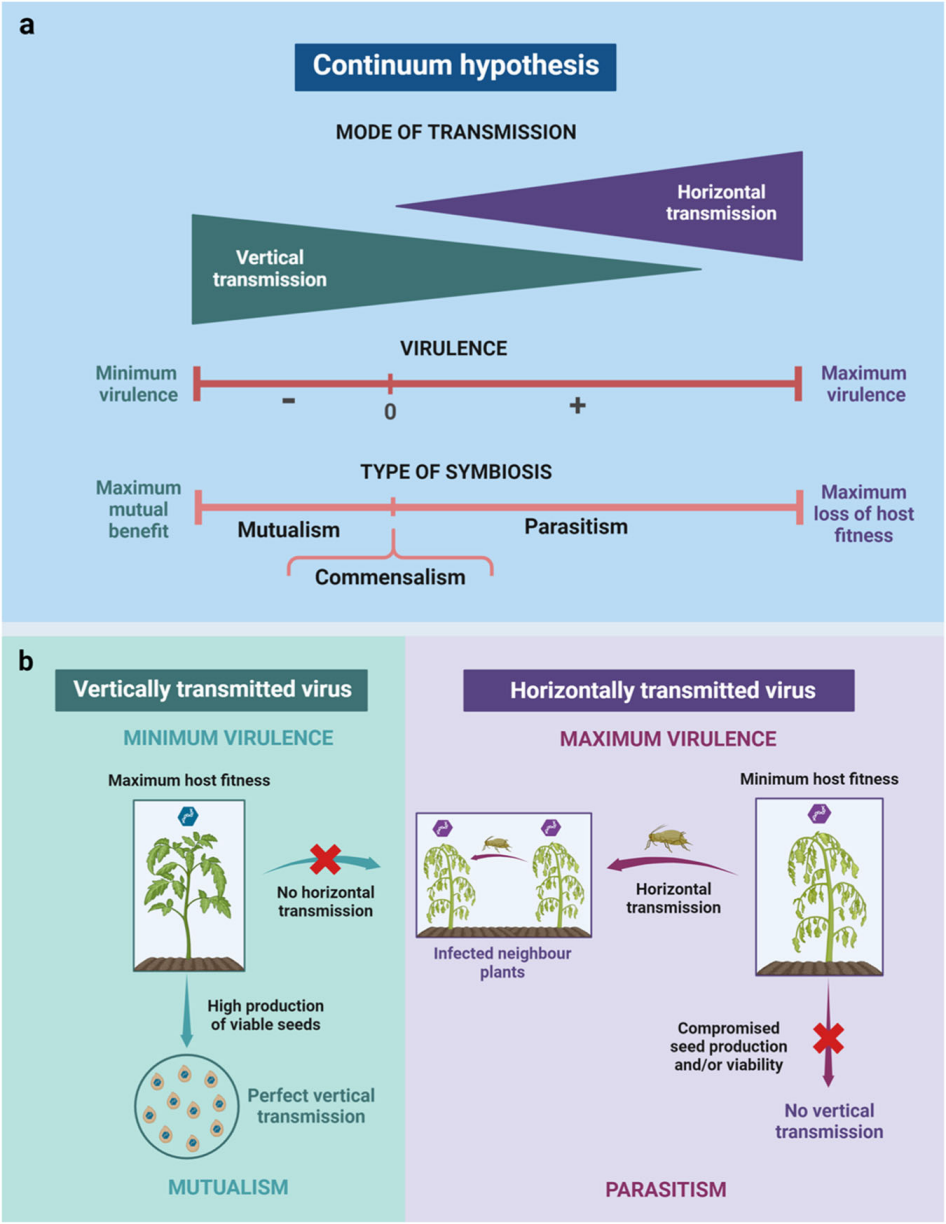
Scheme of the *Continuum hypothesis* adapted to plant viruses from the model developed by ref. ^38^. **a** Relationship between the virus transmission mode (triangles) and virulence (dark red line) leading to a parasitism-commensalism-mutualism continuum (light red line). The width of the triangles represents the relative importance of vertical and horizontal transmission for virus fitness. Mostly vertically transmitted viruses evolve lower virulence to maximize plant fitness and the production of virus-infected seeds. Therefore, these viruses establish more frequently commensalist or mutualistic relationships with the host. In contrast, mostly horizontally transmitted viruses do not require the host to produce progeny and will evolve higher virulence, thus being more often on the parasitism side of the continuum. **b** Illustration of the two extremes of the mutualism-commensalism-parasitism continuum. Left: a strictly vertically transmitted virus will evolve to be avirulent (commensalist) or to confer a benefit to the plant (mutualist), allowing greater production of viable seed and achieving perfect transmission to the plant progeny. Right, a strictly horizontally transmitted virus will maximize virulence (leading to plant castration in the most extreme cases) and, therefore within-host multiplication, which is positively associated with horizontal transmission rate (here represented by the aphid vector). Such viruses will establish a parasitic relationship with the host plant and will not be vertically transmitted.

Using this work as a basis, Ewald^35,38^ proposed that endosymbionts will move along a parasitism-mutualism continuum depending on the relative importance of VT and HT for infection prevalence. That is, parasites would be facultative mutualists depending on the transmission mode (thus the use of the more general term “endosymbiont”), which is referred to as the *Continuum hypothesis* (Fig. 1). According to this hypothesis, the fitness of VT endosymbionts would be highly dependent on host reproductive potential, as such endosymbionts need the hosts to reproduce in order to be transmitted. Thus, such organisms will evolve towards lower (or no) virulence to maximize viable progeny production and, therefore, the number of infected descendants. In contrast, HT endosymbionts will have no direct benefit from increased host fecundity. Following Anderson and May^2^ *Trade-off hypothesis*, Ewald assumed that higher HT is positively correlated with endosymbiont load, which in turn increases virulence. Accordingly, he predicted that HT parasites will tend towards higher virulence to maximize HT (Fig. 1). Let us again use a plant virus with both transmission modes, such as CMV, as an example. According to the *Continuum hypothesis*, a CMV strain in which VT and HT have the same relative importance for its fitness would have intermediate levels of virulence and multiplication. Evolution towards strict VT would reduce virulence to nearly zero. That way, infected plants would produce as many viable seeds as non-infected ones. Even if the percentage of seeds that are infected and can germinate (i.e., infected viable seeds) does not change, reduced virulence would increase the total number of infected propagules produced by the plant, and would therefore be evolutionarily advantageous for the CMV strain adapted to VT. If, additionally, the seed transmission rate evolves to be perfect (100% of infected viable seeds), the evolutionary advantage would be even higher (Fig. 1). This decrease in virulence would be associated with lower virus multiplication, thus coming at the cost of reduced HT. Conversely, for the same CMV isolate with intermediate rates of HT and VT to evolve towards strict HT, an increase in virus multiplication, and therefore in virulence, would be required. This would result in lower production of viable plant seeds and therefore reduced VT.

Yet, virulent parasites with substantial degrees of VT are known to occur (e.g., refs. ^39–41^). Hence, Lipstich et al. ^42,43^ explored the conditions in which VT and high virulence might co-exist. These authors distinguished two epidemiological stages: invasion of the host population and equilibrium. In the former stage, their model predicted that 100% prevalence can only be attained by parasites with both modes of transmission, but not by strict VT or strict HT, providing another evolutionary outcome compatible with the observation that in many plant viruses, both modes of transmission co-exist. At equilibrium, strict VT can maintain 100% prevalence^42^. In this phase, whether VT parasites outcompete HT ones would depend on the level of virulence necessary for parent-to-offspring transmission: High VT attained at low virulence has low cost for host progeny production, which makes this mode of transmission highly efficient. In this context, HT is not favored even if it is highly efficient because as prevalence increases, the number of available hosts for HT decreases, a limitation that does not apply to VT parasites. When high VT requires high virulence, HT is favored as high virulence results in a low number of VT-infected progeny, and this mode of transmission becomes less efficient. Thus, Lipstich and coworkers´ model predicted that the *Continuum hypothesis* only holds if no constraint of high virulence for VT is imposed on the parasite^43^. Using a discrete-time model to consider the dynamics of three different kinds of hosts: uninfected, infected via HT and infected via VT, Lively^44^ reached a similar conclusion. Moreover, this author found that VT parasites could not only establish a mutualistic relationship with the host by evolving to lower virulence, but also by protecting the host from infection by highly virulent HT parasites (provided that VT and HT mixed infections cannot occur).

**Fig. 2:**
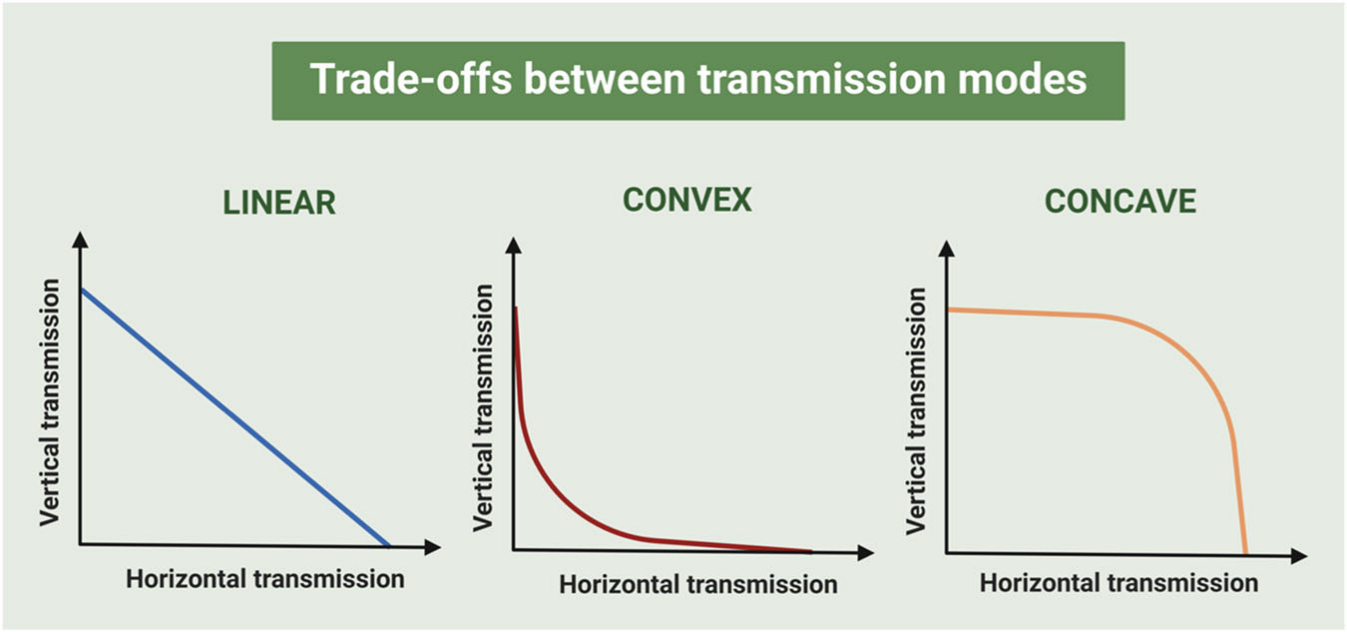
Different types of vertical to horizontal transmission trade-offs. Left: linear trade-off, meaning that vertical transmission rate decreases monotonically as horizontal transmission increases and vice versa. Middle: convex trade-off, meaning that small increases of horizontal transmission lead to a rapid decrease of vertical transmission up to a certain point in which further increases of horizontal transmission have little effect on vertical transmission. Right: concave trade-off, meaning that only large increases in horizontal transmission led to reductions in vertical transmission. For convex and concave trade-offs, extreme cases are represented. Changes in the slope of the correlation would smooth the described effects.

Interestingly, all these seminal models, although intended to be applicable to parasites at large, did not consider a recovery class of hosts such that they are suitable for plant viruses, as plants cannot clear the infection. Thus, using this theoretical work as a basis, plant virus-specific models have been subsequently developed. Indeed, many of them assume a trade-off between VT and HT such that both modes of transmission cannot be simultaneously maximized as posed by the *Continuum hypothesis*. For instance, Hamelin et al.^45^ predicted that, if VT is perfect and the trade-off between modes of transmission is convex (Fig. 2), evolutionary branching results in the appearance of genotypes with either VT or HT. If the trade-off is concave (Fig. 2), intermediate rates of both modes of transmission can co-exist. These predictions are in line with those reported by Bernhauerová and Berec^46^, who addressed the evolution of VT and HT in sexually transmitted parasites considering an additional trade-off with host mortality. In addition, the model developed by Hamelin et al.^45^ predicted that tolerance to virus infection, defined as the ability of the plant to reduce the effect of infection on plant fitness at a given parasite load^47^, selects for high VT. This makes sense as tolerant plants tend to produce more seeds under infection than non-tolerant ones, even at high virus multiplication rates, increasing the advantage of evolving VT. Later, the same authors expanded their model to include the possibility that virus infection increased plant fitness (i.e., the evolution of mutualism from parasitism) and trade-offs of VT and HT with infected host fecundity (i.e., with virulence). Their simulations predicted that, when a VT to virulence trade-off is included, evolution maximizes VT relative to virulence, except if initial virulence is too high, which leads to VT virus extinction. When an HT-to-virulence trade-off was included, both co-existence of parasitic and mutualistic viruses, and the extinction of the parasitic virus leading to strict VT, were possible. This latter outcome leads to mutualists outcompeting parasites^48^.

The vast majority of the theoretical developments discussed above focused on how VT and HT evolve in relation to virulence, considering that all these traits are determined by the parasite. However, there is also a group of models aimed at understanding how host evolution, parasite-specific characteristics or environmental factors affect the evolution of the transmission mode. Yamamura^49^ allowed both the host and the parasite to control the evolution of VT. His simulations predicted that, below a threshold of VT, host and parasite interests are not aligned: the host will evolve towards lower parasite exploitation to reduce the effect of infection on its fitness, and the parasite would evolve towards the opposite as the most important transmission mode below the threshold is HT, which requires higher host exploitation (note that here exploitation might be considered a proxy of parasite multiplication/virulence). In this situation, if the host dominates the interaction, the resulting evolutionary outcome is parasitism, and therefore higher virulence, even if VT exists. If the parasite dominates the interaction during evolution, it is possible that, at some point, a mutation appears that increases VT above the threshold. Then, VT becomes more important than HT and, therefore central for parasite fitness. Consequently, the parasite benefits from suppressing host exploitation (lower virulence) to achieve higher VT. In this context, the interests of parasites and hosts align, leading to co-evolution towards mutualism. Similarly, Shillock et al.^50^ used a game-theory model of co-evolution between parasites and hosts but, at odds with Yamamura^49^, they showed that high VT does not always lead to more benign parasites, for instance, when achieving VT requires high virulence. Bergstrom et al.^6^ explored how transmission bottlenecks affect the evolution of virulence in VT and HT RNA viruses (which is the case for most plant viruses^51^). Their model predicted that stronger population bottlenecks reduce virulence faster in VT than in HT viruses because the stochastic loss (genetic drift) of the most virulent variants in the virus population is more likely in VT virus populations. Also, increasing replication time favours virulence in both VT and HT parasites because it increases the weight of selection and reduces that of bottleneck-associated genetic drift, therefore limiting the loss of virulent variants. The effect of the spatial structure of the host population on the evolution of the transmission mode has also been modeled, predicting that a spatial structure favors VT and reduces virulence due to the limited number of susceptible hosts for HT. In contrast, in well-mixed populations, HT is the preferred strategy as it is more likely to find susceptible hosts^52^. If we use again as an example a plant virus that has both non-persistent HT by vectors and VT via seed such as CMV, the virus would be more likely to be seed transmitted if the plant population is patched, as for HT the vector would need to travel relatively long distances to find another patch of susceptible plants with the risk of becoming non-viruliferous in the meantime. This limitation would not apply to a homogeneous host population. This prediction is in line with that of van den Bosch et al.^53^ using a model specifically developed for fungal plant parasites. Finally, the consequences for plant virus prevalence in the plant population of vertical transovarial transmission in insect vectors have also been modeled^54,55^. Note that these viruses are transmitted in a persistent replicative manner between plants, but generally are not seed transmitted^56^. The model predicted that transovarial transmission increases the number of viruliferous vectors but has little effect on virus prevalence in the plant population. The authors concluded that, despite such limited impact, transovarial virus transmission in the vector is still evolutionarily advantageous as it allows virus persistence when plant hosts are not available.

From the theoretical framework summarized above, two general predictions arise: (i) evolution of VT is generally associated with reduced virulence (although certain conditions may break such relationship), and (ii) maximization of VT or HT is possible, but rarely both transmission modes can be maximized at the same time (perhaps with the exception of infections in tolerant hosts). Both predictions generally fit with the *Continuum hypothesis*, and in the next section, we discuss the experimental evidence supporting or challenging them.

## Experimental evidence of the theoretical predictions on the evolution of parasite transmission mode

For plant viruses, most of the evidence supporting the predictions of the *Continuum hypothesis* is indirect (Table 1). For instance, persistent (cryptic) viruses (*Partitiviridae*, *Endornaviridae*, *Chrysoviridae*, *Totiviridae*) cause asymptomatic infections and are highly prevalent in wild plant populations^20,57^. These cytoplasmic viruses are not transmitted mechanically or by grafting, and have no biological vectors known^58,59^. Persistent viruses apparently do not move from cell-to-cell^60^. Thus, it is thought that they undergo strict vertical transmission via meiosis^61^, generally at high rates^62,63^. Moreover, it has been suggested that the high prevalence of these viruses is explained because they confer a competitive advantage to the plant in certain situations (for instance, tolerance to abiotic stresses)^63^. Hence, they would represent a paradigmatic example of one extreme of the VT to HT continuum: Strict VT with a mutualistic relationship (Fig. 1). Data on plant viruses that cause acute infections are also compatible with the *Continuum hypothesis*. It has been reported that TuMV isolates with lower virulence in *Arabidopsis thaliana* have higher seed transmission than those causing more severe infections^39,64^. Also, CMV is seed transmitted in wild-type *A. thaliana* genotypes^39^, but not in mutants impaired in autophagy whereby virus multiplication and virulence are significantly higher^65^. In the opposite side of the continuum, BYDV, which is a virus restricted to plant phloem that is strictly HT, often induces severe symptoms in the ~100 wild grasses that it infects^56^. In addition, a relatively mild strain of BYDV (MAV) was displaced by a severe strain (PAV) over a 20-year period in New York state. This shift in strain prevalence appears to reflect differences in HT by aphids^56^. Similar dynamics have been reported for two other aphid-borne viruses: sugarcane mosaic virus (SCMV) and maize dwarf mosaic virus (MDMV)^66^. Indirect evidence suggests a positive association between HT and virulence in plant viruses transmitted by other vectors. In susceptible tomato plants, resistance-breaking (RB) isolates of thrip-transmitted tomato spotted wilt virus (TSWV) have HT rates above 60% and as high as 100%, whereas wild-type (WT) isolates have generally lower HT rates (30 to 80%)^67,68^. Because RB isolates induce symptoms in susceptible and resistant plants and WT strains only in susceptible hosts, RB isolates could be considered as more virulent than WT ones. However, when only susceptible hosts were considered, time to symptom development and severity did not differ between RB and WT isolates despite the increased HT rates of the former, which argues against the generality of the *Continuum hypothesis*. In the same line, though TSWV has not been reported to be seed transmitted (but see ref. ^69^), virus isolates causing asymptomatic infections have been repeatedly reported (e.g., refs. ^70,71^). Other examples challenging the predictions of the *Continuum hypothesis* do exist. Virulent strains of raspberry ringspot virus (RPRSV) multiply and induce systemic symptoms more rapidly, are more competitive, and are transmitted to seeds more frequently than less virulent strains^72^. Also, a comparison of seed transmission rates between CMV strains differing in virulence yielded no significant differences^39^ (Table 1).

**Table 1:** Experimental works allowing direct or indirect testing of the *Continuum hypothesis* and plant-virus interactions

Virus^a^	Host	Evidence^b^	Transmission^c^	Relationships between HT, VT and V^d^	Support for the *Continuum Hypothesis*	References
ArLV-1	Arabidopsis	Plant screening	VT (seed)	VT (+)/V (−)	Yes	^63^
BPEV	Pepper	Plant screening	VT (seed)	VT (+)/V (−)	Yes	^58^
BSMV	Barley	Experimental evolution	VT and HT	HT (+)/V (+) VT (+)/V (−) VT (+)/HT (−)	Yes	^73^
BYDV	Various	Epidemiological data	HT (aphids)	HT (+)/V (+)	Yes	^56^
CMV	Arabidopsis	Experimental evolution	VT and HT	VT (+)/V (−) HT(0)/V(0)	Yes No	^74^
CMV	Bean Cucumber Tomato	Experimental evolution	HT (mechanical)	HT(0)/V (0 or +) HT(0)/V (− or +) HT(0)/V (0 or +)	No No No	^81^
CMV	Arabidopsis	Plant screening	HT (mechanical)	VT (+ or −)/V (+or −)	No	^39^
MDMV	Various	Epidemiological data	HT (aphids)	HT (+)/V (+)	Yes	^66^
PCV-1/PCV-2	Pepper	Plant screening	VT (seed)	VT (+)/V (−)	Yes	^57,59^
PPV	Pea	Experimental evolution	HT (aphids)	HT (+)/V (+)	Yes	^76^
RPRSV	Chickweed	Experimental inoculation	VT (seed)	VT (+)/V (+)	No	^72^
SbDV	Pea	Experimental evolution	HT (aphids)	HT (+)/V (+)	Yes	^75^
SCMV	Various	Epidemiological data	HT (aphids)	HT (+)/V (+)	Yes	^66^
TuMV	Arabidopsis	Experimental evolution	HT (mechanical)	HT (+)/V (+)	Yes	^77–80^
TuMV	Arabidopsis	Plant screening	VT (seed)	VT (+)/V (−)	Yes	^39,64^
TSWV	Tomato	Plant screening	HT (thrips)	HT (+)/V(0)	No	^67^

^a^*ArLV-1* Arabidopsis latent virus 1, *BPEV* Bell pepper endornavirus, *BSMV* Barley stripe mosaic virus *BYDV* Barley yellow dwarf virus, *CMV* Cucumber mosaic virus, *MDMV* Maize dwarf mosaic virus, *PCV-1/PCV-2* Pepper cryptic virus 1 and 2, *PPV* Plum pox virus, RPRSV raspberry ringspot virus, *SbDV* Soybean dwarf virus, *SCMV* sugarcane mosaic virus, *TuMV* Turnip mosaic virus.

^b^Epidemiological data: HT and virulence are quantified in the field; plant screening: a set of infected plants was tested for virus seed transmission and virulence; experimental evolution: serial passage experiments.

^c^HT horizontal transmission, *VT* vertical transmission.

^d^V: Virulence. (+) transmission or virulence increases; (0) transmission or virulence does not change; (−) transmission or virulence decreases.

Formal analyses of the predictions of the *Continuum hypothesis* are scant for plant viruses and generally involve the analysis of the effect of virus adaptation to HT and VT through serial passages (Table 1). Following this experimental evolution approach, Stewart et al.^73^ performed 3–4 serial passages of barley stripe mosaic virus through strict HT and strict VT in barley. Virus adaptation to higher VT resulted in a reduction of virulence, whereas adaptation to higher HT increased virulence, thus supporting the relationship between this trait and the transmission mode predicted by theory. Moreover, adaptation to HT resulted in a reduction in the VT rate, also supporting the prediction that both modes of transmission cannot be simultaneously optimized. In a similar experiment involving CMV and *A. thaliana*, serial passages of strict VT increased the efficiency of this transmission mode and reduced both virulence and within-host multiplication. In contrast, serial passages of HT did not alter any of these two traits^74^. These results again fitted one of the predictions of the *Continuum hypothesis*. However, the seed transmission rate of HT passaged viruses was not determined here, which prevented analyzing VT to HT trade-offs. These authors tested the performance of VT-passaged viruses in seeds obtained before and after serial passages of VT to understand the contribution of host-virus co-evolution to the transmission mode. Remarkably, VT passaged viruses had higher seed transmission in passaged than in ancestral plants, indicating that hosts may also adapt to the virus transmission mode, a possibility largely overlooked by theoretical work (see previous section)^74^. To our knowledge, no other experiment involving adaptation to virus seed transmission has been published to date (Table 1). However, several analyses of plant virus adaptation to HT are available in the literature. For example, serial passages of soybean dwarf virus (SbDV) through aphid transmission in pea resulted in a significant increase in HT rate and symptom severity, here used as a proxy of virulence^75^. Similarly, serial passages of plum pox virus (PPV) in pea via aphids resulted in higher HT and accelerated symptom development^76^. Some experiments have also mimicked HT through aphids using mechanical inoculation. One such work involved 60 serial passages of TuMV HT via mechanical inoculation in *A. thaliana*, which resulted in higher virulence (which should negatively affect VT) but reduced plant mortality (which enlarges the infectious period, thus theoretically favoring HT)^77^. Using the same experimental system, the group of Prof. Elena also observed that serial passages of mechanical inoculation generally increased symptom severity (e.g., refs. ^78,79^). It could be argued that the virus evolution towards higher virulence observed in these works is the consequence of the higher inoculum dose achieved through mechanical inoculation as compared to transmission via aphids, as inoculum dose has been linked to symptom severity^51^. To our knowledge, the only study in which serial passages of horizontal transmission through mechanical inoculation and aphid transmission were performed in parallel was reported by Wallis et al.^76^. These authors showed that, although slower in aphid-transmitted virus lineages, evolution towards faster symptom development and higher virus multiplication occurs regardless of the inoculation mode. Moreover, serial passages of mechanical inoculation do not always lead to increased virulence. For instance, Montes et al.^80^ performed HT serial passages of TuMV through mechanical inoculation in tolerant and non-tolerant *A. thaliana* plants. Evolved viruses reduced virulence per unit of parasite load in the former but not in the latter. Although these authors did not quantify VT of the passaged viruses, their observations were compatible with the Hamelin et al.^45^ model. In addition, serial passages of CMV in beans, cucumber, and tomato did not increase infectivity, which could be considered as a proxy of HT, even when evolution resulted in higher virulence^81^ (Table 1). Thus, it is reasonable to conclude that the results of the works referenced here are, at least in part, associated with adaptation to the transmission mode. Overall, 74% of the direct and indirect experimental analyses support the predictions of the *Continuum hypothesis* (Table 1). Although this may appear to be a high percentage, it is based only on ~25 works (many of which are not intended to test this hypothesis), and its generality must be taken with caution.

Although out of the scope of this review, it is worth mentioning that evidence supporting the predictions of the *Continuum hypothesis* also comes from other host-virus interactions. For instance, as early as the 1930s, in an experimental study of the epidemiology of lymphocytic choriomeningitis virus (LCMV) in laboratory mice, in which infected animals were placed in cages with initially uninfected ones, the prevalence of the infection reached 100%, at which point all transmission was vertical. Concurrently, the virulence characteristics of the virus changed, such that infections acquired vertically that had caused 100% morbidity at the beginning of the experiment were asymptomatic by the end^82,83^. The lower morbidity was not due to the development of a stronger immune response in the host because of recurrent exposure to the virus, as the author reported that the absence of disease occurred even if the virus load remained unchanged^83^. Insect viruses have also been shown to display similar patterns, for instance, nuclear polyhedrosis virus in fall armyworm (*Spodoptera frugiperda*)^84^ or acute paralysis virus and deformed wing virus in honeybees^85^. Finally, theoretical predictions on the role of host population spatial structure on the evolution of transmission mode have been supported by experimental analyses in bacteria-phage interactions^52,86^. However, trade-offs between transmission modes have been proven not to be universal: Positive correlations between VT and virus replication/virulence have also been reported for HIV or human papillomavirus^87,88^.

## Determinants of vertical and horizontal transmission

Both theoretical models and experimental evidence seem to largely agree that VT to HT trade-offs mediated by virus multiplication and virulence are widespread in plant-virus (and host-virus) interactions. However, very little is known about the virus genetic determinants of seed transmission, which hampers addressing the molecular bases of the adaptation to the transmission mode^19^. We envision two possibilities that may explain the trade-off between transmission modes:

First, VT and HT genetic determinants are located in different viral proteins. It is commonly acknowledged that the rate of protein evolution is largely set by the fraction of sites that are involved in protein function (i.e., “functional density”)^89,90^. Thus, when mutations in one protein increase VT such that it becomes the main mode of transmission, HT determinants become less functionally relevant, and mutations reducing the HT rate are more likely to accumulate as they will have a lesser effect on virus fitness. Current information on the VT and HT determinants of CMV would fit with this possibility: CMV genetic determinants of seed transmission have been mapped in the viral replicase^91^, whereas those associated with aphid transmission are located in the coat protein^92^. Interestingly, these proteins modulate virus multiplication and symptom development^93^, perhaps explaining with these two traits are associated with the evolution of the transmission mode.

Second, VT and HT genetic determinants co-occur in the same protein or are even the same, and this protein mediates virus multiplication and/or virulence. Thus, mutations have one-way effects increasing or decreasing these traits, such that only one mode of transmission can be maximized, but not both. Examples that may be compatible with this possibility are the species in the genus *Potyvirus*, for which determinants of both HT and VT have been mapped in the helper component proteinase (HC-Pro)^94,95^. The HC-Pro is the viral suppressor of the RNA silencing plant defense response and interacts with the plant RNA silencing machinery and with other components of the plant defenses such as the proteasome^96^. Thus, mutations in protein domains affecting VT and HT likely affect virus multiplication and virulence. Indeed, the HC-Pro domain responsible for the interaction with the proteasome fully overlaps with the region of the HC-Pro where the HT determinant is located^94,97^. Moreover, components of the plant RNA silencing machinery mediate plant meristem invasion by TuMV^98^, which is thought to be key for VT^99^.

To our knowledge, none of these possibilities has been tested, neither others that may exist. Thus, how the VT to HT trade-off is genetically controlled remains unknown.

## Implications of the evolution of parasite transmission mode for the management of virus outbreaks

As mentioned above, the main mode of plant virus transmission is horizontal. In previous sections, we summarized experimental evidence of how increased HT relates to higher per-plant virulence. In addition, from an epidemiological perspective, evolution towards higher HT increases virus prevalence and, therefore virulence at the population levels: The higher the prevalence, the more individuals are infected and the lower the sum of the number of seeds produced by all individuals in the populations^100^. Therefore, research on control methods for HT plant viruses is a hot topic in plant virology. Many of these are directed towards interfering with the vector transmission in different ways. Because several comprehensive reviews dealt with this subject in the past years (e.g., refs. ^101,102^) we will not enter in detail here. However, it is worth mentioning that, in the context of the *Continuum hypothesis*, if these control methods are successful, they will impose selection pressure on the virus for evolving VT, as this becomes the only mode for transmission available. However, to our knowledge, the consequences of control methods on vector transmission for the evolution of VT and virulence have not been experimentally analyzed.

On the other extreme of the HT to VT continuum, seed transmission has far-reaching consequences for plant virus epidemiology. First, seed infection provides the virus with a means to persist for long periods of time when hosts and/or vectors are not available. Second, seed transmission allows for long-distance dissemination of plant viruses, even at a transcontinental scale. Finally, seed transmission is an important source of primary inoculum for many viruses with vertical transmission, which are horizontally disseminated afterwards via vectors^19^. Therefore, virus evolution towards higher VT may cause devastating epidemics. For instance, seed-borne CMV epidemics in pepper, a host in which virus seed infection is high^24^, resulted in yield losses of over 80%^103^. Also, seed-borne alfalfa mosaic virus (AMV) epidemics in Australian pulse crops resulted in yield losses of up to 100%^104^. These epidemics are more devastating at higher VT rates^19,104^. Despite the importance of seed-borne virus outbreaks, control measures are largely limited to seed health tests^19^. The use of certified virus-free seeds or of varieties resistant to seed transmission has also been proposed in a series of theoretical works on Cassava mosaic disease (CMD) and Maize lethal necrosis (MLN), which are caused by a combination of plant viruses^105–107^. These models predict that the use of clean seeds may result in virus eradication, provided that the economic cost of these seeds is not high. Interestingly, combining resistance to seed transmission and the use of clean seeds may be counterintuitive, reverting the beneficial effect of using clean seeds in controlling disease epidemics. This is because, in this context the use of clean seeds becomes not profitable as reduced VT due to plant resistance does not justify the seed cost. Indeed, currently, the use of clean seeds, but not of resistance to seed transmission, is advised in countries where CMD and MLN are endemic^108^. Again, the consequences of reducing VT for the evolution of HT remain unexplored.

## Concluding remarks

The capacity of a parasite to be transmitted to new susceptible hosts determines the severity and persistence of epidemics. Therefore, understanding how parasites evolve and optimize different modes of transmission has received considerable attention in the past decades from a theoretical perspective. The *Continuum hypothesis* results from such efforts and provides a conceptual framework to explore how VT and HT are optimized and the factors affecting this process. Theoretical developments have not been accompanied by a similar experimental effort to test the predictions of the *Continuum hypothesis* (Table 1). Consequently, the generality of the predictions of the *Continuum hypothesis* remains debatable, and various aspects of the conditions promoting one or the other mode of transmission, as well as the molecular basis controlling the evolution of VT and HT, remain poorly understood. We think that future studies would pay special attention to aspects that include, but are not restricted to:

- Despite the ample use of mathematical modelling to explore the conditions in which HT and/or VT evolve in plant-virus interactions, most of these models only let the parasite evolve. However, experimental evidence points to a relevant role of host evolution in determining the mode of transmission, such that parasite evolution is not the only force at play^74^. Considering how co-evolutionary processes may influence the optimization of VT and HT will yield valuable information on the evolution of parasite transmission mode. Indeed, the few theoretical works allowing for host-parasite co-evolution expand the array of evolutionary outcomes regarding the parasite transmission mode as compared with models exclusively considering virus evolution. In addition, plant viruses are, in general, multi-host parasites. For instance, CMV can infect more than 1000 plant species^93^. However, most mathematical models on the evolution of the transmission mode consider one parasite in a single host. Between-host adaptation trade-offs have been described for plant viruses^109^, such that changes that allow plant virus adaptation to VT in a given host would not be universal to others. Therefore, an effort should be made to develop a theoretical framework to include the evolution of parasites in more than one host. For this purpose, models that explore the prevalence of plant viruses considering more than one host genotype and both VT and HT exist^110^, and could be adapted by incorporating the modelling of between-host fitness trade-offs.
- The *Continuum hypothesis* was formulated more than 30 years ago. Still, the experimental evidence supporting or challenging this hypothesis is scant, at least for plant-virus interactions. Addressing its generality requires expanding the number of pathosystems currently analyzed. Experimental evolution experiments are a suitable approach for this purpose. Certainly, serial passages of VT may be time-consuming if long-lived plant hosts are utilized. Using short-lived hosts, such as *A. thaliana*, maybe a way of overcoming this limitation. It is also worth mentioning that, to date, most experimental analyses on the evolution of the transmission mode utilized serial passages of mechanical inoculation as a proxy of HT. Considering that in nature, many plant viruses are transmitted through aphids, using the actual vector in serial passage experiments would be a more realistic way of testing the *Continuum hypothesis*.
- A main premise of many mathematical models on the evolution of the parasite mode of transmission is that there is a trade-off between VT and HT. In general, the few experimental analyses of the *Continuum hypothesis* that involved parallel evolution of the same virus strain by strict VT and strict HT quantified VT only in the viruses evolved through seed transmission (but see ref. ^73^). Moreover, none considered analyzing HT rate of the isolates passaged by VT. Therefore, future studies should address whether the VT to HT trade-off holds in plant-virus interactions. This would require performing serial passages of strict HT and strict VT of the same virus isolate in parallel, and further analyses of the efficiency of both modes of transmission in the evolved viruses.
- There is very little information on the molecular bases of plant virus adaptation to a given transmission mode. For instance, none of the serial passage experiments described in this review mapped the genomic changes associated with modifications of VT. Only some of the experiments in which HT passages were performed did so. With the development of next-generation sequencing techniques, obtaining sequence information has become easier and more affordable. Incorporating this information into experimental evolution approaches will provide a more comprehensive picture of how viruses evolve, one of the main traits that control their fitness.
- Finally, there is a lack of information on how the evolution of plant virus transmission mode relates to the management of viral epidemics in field conditions. Approaching this problem would require understanding the relative contribution of VT and HT to virus fitness before and after applying control measures. This information is currently lacking for virtually every plant virus^19^. In addition, in nature mixed infections by more than one plant virus in the same plant are commonplace^111^. Co-existing viruses may also share vectors and seeds as vehicles for transmission. Thus, how virus-virus antagonistic and synergistic interactions affect the evolution of the transmission mode in the presence and absence of control methods should also be addressed.
